# Predicting Gait Speed Improvement in Idiopathic Normal Pressure Hydrocephalus Patients: The Role of Evans Index and Ventricular Volume

**DOI:** 10.7759/cureus.61799

**Published:** 2024-06-06

**Authors:** Saurabh Rohatgi, Amol Dua, Arman Avesta, Rehab Naeem Khalid, Andrea Diociasi, Karen Buch, Jeremy N Ford, Rajiv Gupta

**Affiliations:** 1 Department of Radiology, Division of Neuroradiology, Massachusetts General Hospital, Harvard Medical School, Boston, USA

**Keywords:** normal pressure hydrocephalus, gait speed, cerebrospinal fluid (csf), lumbar drain, lumbar puncture, ventricular volume, evans index (ei)

## Abstract

Objective

This study aims to investigate the association between specific imaging parameters, namely, the Evans index (EI) and ventricular volume (VV), and the variation in gait speed observed in patients with idiopathic normal pressure hydrocephalus (iNPH) before and after cerebrospinal fluid (CSF) removal/lumbar drain (LD). Furthermore, it seeks to identify which imaging parameters are the most reliable predictors for significant improvements in gait speed post procedure.

Methods

In this retrospective analysis, the study measured the gait speed of 35 patients diagnosed with idiopathic normal pressure hydrocephalus (iNPH) before and after they underwent CSF removal. Before lumbar drain (LD), brain images were segmented to calculate the Evans index and ventricular volume. The study explored the relationship between these imaging parameters (the Evans index and ventricular volume) and the improvement in gait speed following CSF removal. Patients were divided into two categories based on the degree of improvement in gait speed, and we compared the imaging parameters between these groups. Receiver operating characteristic (ROC) curve analysis was employed to determine the optimal imaging parameter thresholds predictive of gait speed enhancement. Finally, the study assessed the predictive accuracy of these thresholds for identifying patients likely to experience improved gait speed post-LD.

Results

Following CSF removal/lumbar drain, the participants significantly improved in gait speed, as indicated by a paired sample t-test (p-value = 0.0017). A moderate positive correlation was observed between the imaging parameters (EI and VV) and the improvement in gait speed post-LD. Significant differences were detected between the two patient groups regarding EI, VV, and a composite score (statistical test value = 3.1, 2.8, and 2.9, respectively; p-value < 0.01). Receiver operating characteristic (ROC) curve analysis identified the optimal thresholds for the EI and VV to be 0.39 and 110.78 cm³, respectively. The classification based on these thresholds yielded significant associations between patients displaying favorable imaging parameters and those demonstrating improved gait speed post-LD, with chi-square (χ²) values of 8.5 and 7.1, respectively, and p-values < 0.01. Furthermore, these imaging parameter thresholds had a 74% accuracy rate in predicting patients who would improve post-LD.

Conclusion

The study demonstrates that ventricle volume and the Evans index can significantly predict gait speed improvement after lumbar drain (LD) in patients with iNPH.

## Introduction

Idiopathic normal pressure hydrocephalus (iNPH) is a neurological condition in which the ventricles are dilated with normal cerebrospinal fluid (CSF) pressure [1]. The prevalence of iNPH reported in various studies varies between 0.1% and 2.9% [2], with the incidence increasing to 5.9% in patients 80 years and older [3]. This condition is characterized by a triad of symptoms: cognitive decline, urinary incontinence, and gait disturbances [4,5]. Among these symptoms, gait disturbances have been recognized as the initial and hallmark manifestation of iNPH [6-8]. Clinically, the iNPH gait is described as a hypokinetic gait with reduced gait speed, diminished stride length due to the simultaneous contraction of proximal muscles, reduced step height, and dynamic disequilibrium [2,8].

Lumbar puncture (LP) is a valuable diagnostic tool in diagnosing and managing iNPH [6,9]. Given that gait impairment is one of the hallmark manifestations of iNPH, the clinical significance of quantifying gait speed and estimating the degree of improvement post lumbar puncture using several different radiological parameters is crucial in managing these patients. A previous literature review revealed various studies exploring several independent factors and their association with iNPH and investigating gait changes [2,10]. Most studies on gait changes after lumbar puncture use clinical gait scores compared to quantitative data [11,12]. The purposes of our study were primarily to observe the interplay between gait speed variation after lumbar drain (LD) and pre-LD radiological parameters (the Evans index {EI} and ventricular volume {VV}) in patients with idiopathic normal pressure hydrocephalus (iNPH), aiming to determine threshold values associated with the most significant improvement in gait speed. Additionally, the current study incorporates a combined composite score that utilizes both the ventricular volume and Evans index to further understand their relationship with gait parameters.

## Materials and methods

This study was approved by the institutional review board of Massachusetts General Hospital (approval number: 2022P001900) and conducted at an academic medical center. Magnetic resonance (MR) or computed tomography (CT) scans before LP were obtained using a keyword database search in the Research Patient Data Registry. We looked for "idiopathic normal pressure hydrocephalus (iNPH) and IR lumbar puncture diagnostic (test: IRP.NSN.LMPDX)" to select patients for the study.

The current study identified 42 patients with the clinical diagnosis of iNPH. The inclusion criteria included patients with the clinical diagnostic criteria for iNPH undergoing lumbar puncture. The exclusion criteria included the inability to undergo CSF drainage or complete a gait analysis post-CSF drainage. Seven patients were excluded from the study based on these exclusion criteria. Thirty-five patients were eligible for statistical analysis, including 16 males and 19 females, with a mean age of 82 years and a standard deviation (SD) of 2.

The physical therapy department conducted a clinical assessment of quantitative gait analysis. Gait analysis was done before LP and three days after CSF removal/LD. Gait analysis comprised parameters including, but not limited to, cadence, gait speed, stride length, timed up and go, and balance. For this study, information regarding gait speed was extracted from the gait analysis reports. Gait speed refers to the distance (meters) covered every second.

**Figure 1:**
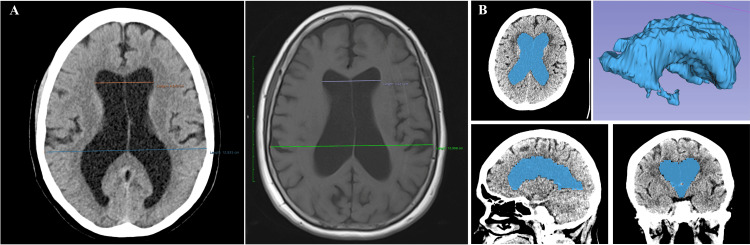
Axial image measuring the Evans index as the ratio between the frontal horns' maximum width and the cranium's maximum inner diameter on CT and T1-weighted images (A). CT image showing the segmentation of the ventricular volume shaded in blue on axial, sagittal, and coronal images with final segmented output (B). CT: computed tomography

A radiological assessment of the Evans index (EI) was performed using CT and 3D T1-weighted MR images. The EI was calculated in the axial image as the ratio between the lateral ventricles' frontal horns and the skull's maximum inner diameter in the same slice (2). To compute the ventricular volume, we manually segmented the lateral and third ventricles using 3D Slicer (version 5.2.2, http://www.slicer.org) and then computed the ventricular volume (cm³) (Figure 1).

Board-certified radiologists independently assessed the images. A combined "composite imaging score" (composite score) was computed by averaging the normalized ventricular volume and the normalized EI. Normalization was done using a Z-score: the mean and standard deviation for each parameter were computed for the entire cohort of patients. The Z-score for each patient was computed by subtracting the mean from each patient's value and dividing the resultant difference by the standard deviation.

Statistical analysis

Gait speed obtained before LP was compared with that obtained three days after CSF removal/LD, using the paired t-test. Furthermore, the study measured the correlation between the EI, ventricle volume, and a composite score with a change in gait speed after LD using Spearman's correlation. Once correlation was established, the study divided the patients into two groups based on thresholding the gait speed using a Z-score of 0.1. Differences between the two groups regarding the EI, ventricle volume, and a composite score were explored using the Wilcoxon rank-sum test. Finally, to determine which volume and EI measurement exhibited the strongest correlation with gait speed status, a receiver operating characteristic (ROC) analysis was employed. A chi-square (χ²) analysis was also conducted specifically for the identified volume (110.78 cm³) and EI (0.389). An accuracy analysis was performed using the determined thresholds to strengthen the results further. This comprehensive approach allowed for a thorough examination of the associations between the selected variables and gait speed status in the study. A significance threshold was set at p-value < 0.05. All our statistical evaluations were conducted in the Spyder environment (https://www.spyder-ide.org), compatible with Python 3.10.

## Results

The sample of 35 patients showed a mean ventricular volume of 145 cm³, with a standard deviation of 20. The mean EI was 0.38, with a 95% confidence interval of 0.37-0.39 (Table 1).

**Table 1:** Demographic characteristics, the Evans index, ventricular volume, and gait speed response. LP, lumbar puncture; LD, lumbar drain

	Values
Mean age (years)	82.26 (±80.45-84.06)
Males	45.71%
Females	54.29%
Mean ventricular volume (cm³)	145.28 (±124.77-165.79)
Mean Evans index	0.38 (±0.37-0.39)
Mean gait speed before LP/LD (m/second)	0.51
Mean gait speed after LD (m/second)	0.63
Patients showing gait speed improvement	74.3%
Patients showing no improvement	25.7%

**Figure 2:**
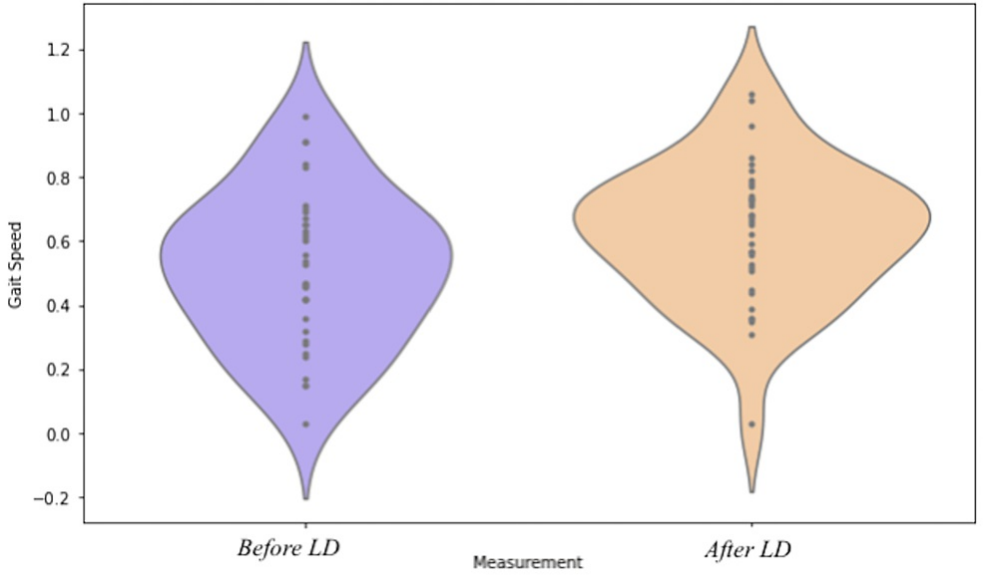
Violin plot comparing gait speed before and after LD (purple represents before LD and orange after LD). LD: lumbar drain

The participants' gait speed was assessed before and after the LD. Before LD, the mean gait speed was 0.51 m/second. After LD, gait speed significantly increased to a mean of 0.63 m/second (paired sample t-test p-value of 0.0017) (Figure 2), a relative gait speed improvement of 23%. Among the 35 participants, 26 (74%) exhibited an improvement in gait speed post-LD, while the remaining nine (26%) showed no significant change (see Table 1).

**Figure 3:**
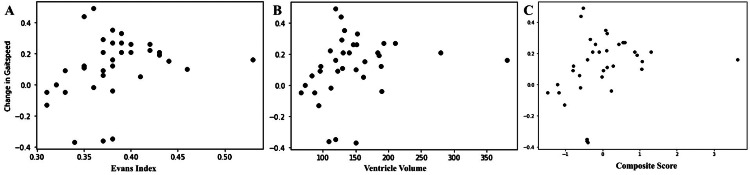
Spearman's correlation for change in gait speed with the Evans index (A), ventricle volume (B), and composite score (C).

Spearman's correlation analysis was employed to investigate the association between specific imaging parameters and changes in gait speed after LD. This analysis indicated a moderate positive relationship between changes in gait speed and radiological parameter results: the EI had a correlation coefficient of 0.400 (p-value of 0.017), ventricular volume had a correlation coefficient of 0.401 (p-value of 0.016), and the composite score (of volume and the EI) had a correlation coefficient of 0.411 (p-value of 0.014) (Figure 3).

**Figure 4:**
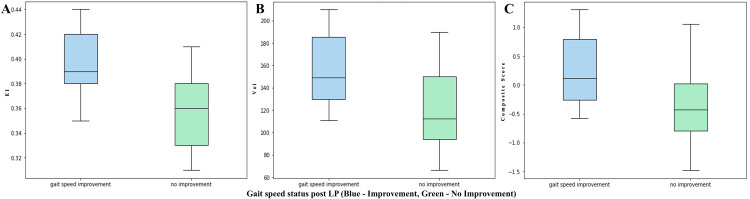
Wilcoxon rank-sum test for (A) the Evans index (EI), (B) ventricle volume, and (C) composite score. LP: lumbar puncture

The study found a significant association between two groups of patients, categorized based on a Z-score threshold of 0.1, and imaging parameters (Figure 4), including the EI (Wilcoxon rank-sum test statistic, 3.1; p-value, 0.0015), ventricular volume (Wilcoxon rank-sum test statistic, 2.8; p-value, 0.005), and a composite score of volume and the EI (Wilcoxon rank-sum test statistic, 2.9; p-value, 0.004). These findings suggest substantial variances in pre-LP/LD EI, ventricular volume, and a composite score of volume and the EI between the participants who experienced gait speed enhancements post-LD and those who did not.

**Figure 5:**
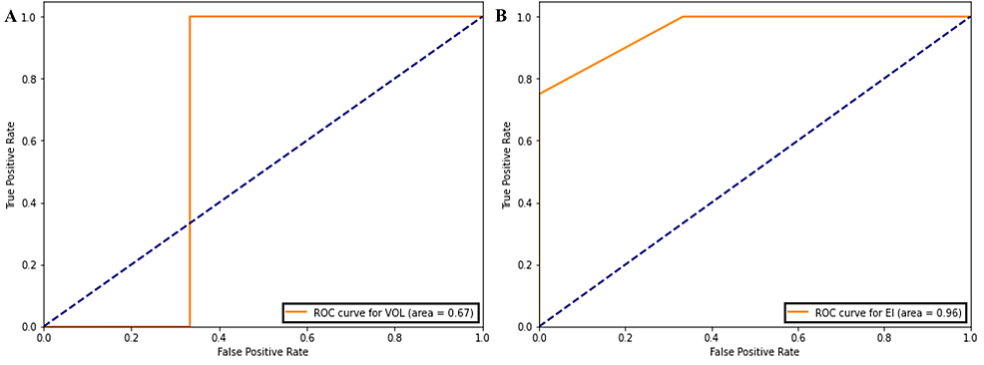
Receiver operating characteristic (ROC) curve analysis for (A) ventricle volume threshold, 110.78, and (B) EI threshold, 0.389. EI: Evans index

The ROC curve analysis (Figure 5) showed an optimal ventricular volume threshold of 110.78 cm³, and its significance was validated via the chi-square analysis (χ² = 8.474; p-value = 0.003). Similarly, an optimal EI threshold of 0.389 was found (χ² = 7.128; p-value = 0.0076), indicating a significant difference in gait speed improvement beyond this threshold. By thresholding the ventricular volume and EI, imaging parameters predict response to LD with 74% accuracy.

## Discussion

Gait impairment is one of the earliest and hallmark manifestations of iNPH [13-15], with gait abnormalities and analyses assessed with numerous studies in the literature with iNPH [4,13,14]. Given that gait speed has been described as an essential feature of mobility impairment and quality of life [2], the current study focused on gait speed and its correlation with radiological parameters such as ventricular volume, the EI, and a composite score of ventricular volume and the EI aiming to determine threshold values associated with a significant improvement in gait speed.

Lumbar puncture is an essential aspect of the diagnostic criteria [6] and a valuable intervention for managing iNPH [9]. Gait speed typically shows the most improvement after CSF removal [16]. The present study confirms this result, demonstrating a notable improvement in gait speed among patients after lumbar drainage: 26 out of 35 patients showed an improvement in gait speed, corresponding to a 23.5% increase in the average gait speed following LD (Figure 2). Similar results in a comparable improvement in gait velocity were also reported by Stolze et al. [4] and Bovonsunthonchai et al. [17]. This change was statistically significant and reaffirmed the impact of cerebrospinal fluid drainage on the motor function of iNPH patients [8,9].

The findings of the correlation analysis shed light on the relationship between alterations in gait speed and radiological parameters. The results unveiled a moderate positive correlation between changes in gait speed and specific radiological measures. Notably, the Evans index (EI), ventricular volume, and the composite score, derived from both ventricular volume and the EI, demonstrated a moderately significant correlation (p-value < 0.05). These findings underscore the potential significance of radiological parameters in understanding variations in gait speed. The current findings are consistent with previous research by Schniepp et al., who identified a positive correlation between the EI and early improvements in walking speed [2].

Using the Wilcoxon rank-sum test, significant disparities were observed across distinct patient cohorts. The current study categorized the study group according to a specific Z-score threshold of 0.1. This separation allowed us to visualize clear patterns in the radiological parameters between individuals who experienced enhancements in gait speed following lumbar drain (LD) and those who did not observe such improvements. The implication is that specific baseline characteristics, as reflected in radiological measures, may serve as prognostic indicators for assessing the likelihood of gait improvement post-LP/LD intervention.

Moreover, when we looked at the data using ROC curve analysis, the study found specific levels of ventricular volume (>110.78 cm³) and the EI (>0.389) that were linked to significant improvements in gait speed after LD. This observation was confirmed by chi-square analysis, showing that gait speed improved notably beyond these thresholds. The accuracy of this model in predicting gait speed improvement after LD was around 74.29%, suggesting its potential usefulness in clinical practice. A recent study using automated segmentation with volumetric analysis also concluded that it could reliably predict shunt responders with an accuracy of 0.90% [18]. However, a retrospective study by Carlsen et al. used a normal pressure hydrocephalus Radscale that combined seven different structural imaging markers to assess shunt responders from non-responders, which only showed moderate discrimination for shunt response [19].

These findings can guide clinicians in making decisions when managing patients with iNPH. By identifying ventricular volume and the EI as possible indicators of gait speed improvement after LP/LD, the present study suggests the possibility of creating more detailed models that consider both clinical symptoms and radiological measurements.

However, it is essential to note some limitations of the current study, including inherent biases given the retrospective nature of the study, a small cohort of patients that limited drawing robust conclusions, longitudinal follow-up in gait improvement not being assessed, our cohort including older patients, and additional comorbid conditions such as Parkinson's and arthritis not being assessed. It would be beneficial to confirm these results with larger datasets. With the advent of artificial intelligence and machine learning frameworks, we can better harness data time points based on the clinical history and symptomology with more robust screening tools based on structural imaging.

## Conclusions

The study demonstrates that ventricle volume and Evans index thresholds can predict gait speed improvement in patients following CSF removal/LD. These findings may guide more targeted clinical approaches to managing iNPH. Establishing threshold levels heralds the advent of more sophisticated, predictive treatment frameworks for iNPH. By integrating clinical insights with detailed quantitative imaging, this approach promises to significantly refine patient treatment strategies.
